# Substance use, Unlike Dolutegravir, is Associated with Mood Symptoms in People Living with HIV

**DOI:** 10.1007/s10461-021-03272-2

**Published:** 2021-04-27

**Authors:** Lisa Van de Wijer, Wouter van der Heijden, Mike van Verseveld, Mihai Netea, Quirijn de Mast, Arnt Schellekens, André van der Ven

**Affiliations:** 1 Department of Internal Medicine, Radboud University Medical Center, PO Box 9101, 6500 HB Nijmegen, The Netherlands; 2 Nijmegen Institute for Scientist Practitioners in Addiction, Nijmegen, The Netherlands; 3 Department of Psychiatry, Radboud University Medical Center, Nijmegen, The Netherlands; 4 Donders Institute for Brain, Cognition and Behavior, Radboud University Medical Center, Nijmegen, The Netherlands

**Keywords:** Dolutegravir, Side effects, Central nervous system, Depression, Impulsive behavior, Anti-retroviral agents

## Abstract

Contradictory data have been reported concerning neuropsychiatric side effects of the first-line antiretroviral drug dolutegravir, which may be partly due to lack of control groups or psychiatric assessment tools. Using validated self-report questionnaires, we compared mood and anxiety (DASS-42), impulsivity (BIS-11), and substance use (MATE-Q) between dolutegravir-treated and dolutegravir-naive people living with HIV (PLHIV). We analyzed 194, mostly male, PLHIV on long-term treatment of whom 82/194 (42.3%) used dolutegravir for a median (IQR) of 280 (258) days. Overall, 51/194 (26.3%) participants reported DASS-42 scores above the normal cut-off, 27/194 (13.5%) were classified as highly impulsive, and 58/194 (29.9%) regularly used recreational drugs. Regular substance use was positively associated with depression (*p* = *0.012*) and stress scores (*p* = *0.045*). We observed no differences between dolutegravir-treated and dolutegravir-naive PLHIV. Our data show that depressed and anxious moods and impulsivity are common in PLHIV and associate with substance use and not with dolutegravir use.

**Supplementary Information:**

The online version contains supplementary material available at 10.1007/s10461-021-03272-2.

## Introduction

All recent national and international guidelines include the integrase inhibitor (INSTI) dolutegravir (DTG) in the list of first-line antiretroviral drugs (ARVs) for people living with HIV (PLHIV) [1]. DTG has an excellent efficacy, limited drug-drug interactions, a high genetic barrier to resistance and is generally well tolerated [2]. Moreover, generic versions became available for low-and middle income countries with a median price of as low as $75 per year [3]. Contradictory findings have been reported concerning neuropsychiatric side effects of DTG. While early randomized trials reported low discontinuation rates due to side effects among DTG-treated individuals (2–4%) [4–8], several recent observational studies showed rates up to 15% [9–13]. The majority of these side effects involved neuropsychiatric symptoms, including insomnia and depressive symptoms. Data on the prevalence and nature of the neuropsychiatric side effects, as well as associated risk factors (e.g. female sex, older age or concomitant use of the ARV drug abacavir [ABC] [10]) are limited. In addition, it remains unclear whether these neuropsychiatric symptoms are specific for DTG or represent an INSTI class effect [14]. Most currently available studies lacked psychiatric assessment tools or proper control groups and often relied on spontaneous recording of side effects in medical records. In addition, many did not take possible confounders, such as recreational drug use, into account. As mental health and adherence are critical for successful cART, more data on the tolerability of DTG and other INSTI are needed. Using validated psychiatric assessment tools, we compared mood, impulsivity, and recreational substance use between DTG-treated and DTG-naive individuals, as well as between INSTI-treated and non-INSTI treated individuals.

## Methods

This cross-sectional observational study is part of the 200HIV Human Functional Genomics Project (HFGP), which focuses on interactions between genetic variations, microbial communities and phenotypic variation, including mental health (www.humanfunctionalgenomics.org) [15]. The Medical Ethical Review Committee Arnhem-Nijmegen (Ref. 2012–550) approved the study protocol and all participants provided written informed consent. Between December 2015 and February 2017, a consecutive series of PLHIV from the HIV-clinic of Radboud university medical center, Nijmegen, The Netherlands were asked to participate in our study. Caucasian individuals aged ≥ 18 years, on cART ≥ 6 months with HIV-RNA load ≤ 200 copies/mL and without active hepatitis B/C or signs of acute infections, were considered eligible. Here, we analyzed all DTG-treated individuals and DTG-naive individuals from the HFGP. Eight participants were excluded because of prior discontinuation of DTG. These individuals did not differ from the DTG-treated individuals in age, sex, CD4 nadir, or scores on the self-report questionnaires. DTG had been discontinued after a median (IQR) of 111(350) days. In 7/8 (87.5%) patients, possible side effects leading to discontinuation were recorded (multiple reasons could be recorded per patient): sleep disturbances (n = 2), psychiatric symptoms (n = 2), headache (n = 3), fatigue (n = 2), dizziness (n = 1), gastro-intestinal complaints (n = 1), and itch (n = 1).

Socio-demographic information and information on health status were collected using an extensive questionnaire, including questions on recently experienced concentration disturbances and headache complaints. Clinical data were extracted from clinical files.

Mood disorders, anxiety, and substance use are among the most commonly reported psychiatric diagnoses in PLHIV [16]. In addition, we and others observed high levels of impulsivity, translating into risk behavior PLHIV [17–19]. We selected golden standard questionnaires focused on mood and anxiety, impulsivity, and substance use meeting the following conditions: 1. self-reporting 2. available in Dutch 3. maximum length of ten minutes 4. validated in healthy and patient populations. The 42-item version of the Depression Anxiety Stress Scale-42 (DASS-42) measures levels of depression, anxiety and stress, with higher scores indicating higher symptom levels. The DASS-42 has a high internal consistency and reliability, and has been validated in Dutch populations [20, 21]. The Barratt Impulsiveness Scale-11 (BIS-11) assesses overall impulsivity and three subscales – attention, motor and non-planning. Higher scores indicate greater impulsivity [22]. The Dutch version of the BIS-11 has been validated in normal and clinical samples [23]. We used the substance use module of the Dutch version of the Measurements in the Addictions for Triage and Evaluation Questionnaire (MATE-Q), which assesses psychoactive substance use, both in the recent past (≤ 30 days prior study visit) and in lifetime [24]. The MATE-Q is based on the WHO’s Composed International Diagnostic Interview (CIDI, version 2.1) and has been validated in Dutch populations [25].

We conducted a post-hoc power analysis for multivariate analysis of variance (MANOVA) with three dependent variables (DASS-42 or BIS-11 subscales) and two groups (DTG vs. DTG-naive) in G*POWER [26]. The available total sample size of 194 allowed detection of a DTG-effect of f^2^ = 0.06 (considered small-medium) [27], with 80% power and α = 0.05. Comparisons of general characteristics between DTG and DTG-naive individuals were made using Student’s T-test (or Mann–Whitney U) for continuous variables and χ^2^ (or Fisher’s exact) for categorical variables. Differences in DASS-42 and BIS-11 scores between DTG and DTG-naive were analyzed using MANCOVA. In case of missing values of < 15%, values were imputed by pooling the scores of the affected subscale(s). Logistic regression analyses were performed for dichotomous data (regular substance use and DASS-42 or BIS-11 scores above the normal cut-off). Spearman’s correlations were applied to explore associations between substance use, and DASS-42 and BIS-11 scores. Additional MANCOVAs were performed to compare DTG with other INSTI (elvitegravir and raltegravir), INSTI with non-INSTI, and DTG-ABC with DTG-non-ABC. Subgroup analyses were performed for older individuals (age ≥ 55 years), females, and those on non-efavirenz cART (given efavirenz’ association with neuropsychiatric symptoms) [28]. Age, nadir CD4^+^ cell count, and duration of HIV infection were included as covariates in the adjusted models. To avoid inflating the risk of Type 1 errors, only adjusted MANCOVAs with* p* < 0.05 were followed by univariate analyses (with Bonferroni correction). A two-tailed * p* of < 0.05 was considered statistically significant. Data were analyzed using SPSS (SPSS 24, Inc. Chicago, Illinois, USA).

## Results

After exclusion of eight individuals with missing values of ≥ 15% in the DASS-42 or BIS-11, we analyzed 194 individuals of whom 82/194 (42.3%) used DTG-containing cART (Fig. S1 Study Flowchart, electronic supplementary material). Study participants had a median (IQR) age of 52.5 (13.3) years and the majority (176/194 [90.7%]) were male. DTG-treated individuals were slightly younger compared to DTG-naive individuals (*p* = *0.013*) and had a shorter duration of HIV infection (*p* = *0.0024*) and cART (*p* = *0.0015*; Table 1). The median (IQR) duration of DTG treatment was 280 (258) days. DTG was combined with ABC in 59/82 (72.0%) individuals, with emtricitabine-tenofovir in 13/82 (15.9%), with boosted atazanavir or darunavir in 8/82 (9.8%), and as dual therapy with lamivudine in 2/82 (2.4%).

**Table 1:** General characteristics

Characteristic	DTG (n = 82)	DTG-naïve (n = 112)	*p*-value
Age, years	50.5 (17.4)	54.4 (13.7)	0.013
Age ≥ 55 years, n/N (%)	27/81 (32.9)	53/112 (47.3)	0.055
Sex, male, n/N (%)	77/82 (93.9)	99/112 (88.4)	0.22
BMI, kg/m^2^	24.0 (3.6)	24.3 (4.1)	0.87
Time since HIV diagnosis, years	7.1 (6.7)	9.3 (10.4)	0.0024
Nadir CD4^+^ count, cells/μl	270 (230)	225 (240)	0.31
CD4^+^ count, cells/μl	640 (280)	680 (345)	0.18
cART-naive, n/N (%)	11/82 (13.4)	19/112 (17.0)	0.55
Time on cART, years	5.6 (6.4)	7.6 (10.8)	0.0013
Time on DTG, days	280 (258)	–	–
cART backbone, n/N (%)			
Tenofovir-emtricitabine	13/82 (15.9)	72/112 (64.3)	< 0.0001
Abacavir-lamivudine	59/82 (72.0)	25/112 (22.3)	< 0.0001
cART regimen, n/N (%)			
NNRTI	1/82 (1.2)	56/112 (50.0)	< 0.0001
Efavirenz	1/82 (1.2)	16/112 (14.3)	0.0013
PI	8/82 (9.8)	21/112 (18.8)	0.10
INSTI	82/82 (100.0)	50/112 (44.6)	< 0.0001
Raltegravir	–	35/112 (31.3)	–
Elvitegravir	–	14/112 (12.5)	–
Psychofarmaca, n/N (%)	12/82 (14.6)	14/112 (12.5)	0.68
Benzodiazepines	7/82 (8.5)	6/112 (5.4)	0.40
Tricyclic antidepressants	1/82 (1.2)	3/112 (2.7)	0.64
Selective serotonin reuptake inhibitors	5/82 (6.1)	3/112 (2.7)	0.29
Methylphenidate	1/82 (1.2)	1/112 (0.9)	1.00
Other	5/82 (6.1)	7/112 (6.3)	1.00
Reported frequent headache, n/N (%)	11/80 (13.8)	16/109 (14.7)	1.00
Reported concentration problems, n/N (%)	19/80 (23.8)	26/109 (23.9)	1.00

Data depicted as median (IQR) unless stated otherwise. Data analyzed using Mann–Whitney U or χ^2^ (or Fisher’s exact) where applicable

*cART* combination antiretroviral therapy, *DTG* dolutegravir, *NNRTI* non-nucleoside reverse transcriptase inhibitor, *INSTI* integrase inhibitor, *PI* protease inhibitor

Psychiatric symptoms were common in our population. Overall, 51/194 (26.3%) individuals scored above the normal cut-off for depression, anxiety or stress (DASS-42), and 27/194 (13.9%) individuals were classified as highly impulsive (BIS-11 total score ≥ 72) [23]. Scores did not significantly differ between DTG-treated and DTG-naive individuals, after adjustment for age, nadir CD4 + cell count, and duration of HIV infection. Similarly, we observed no differences when comparing DTG (n = 82) with other INSTIs (raltegravir and elvitegravir, n = 49), or INSTIs (n = 132) with non-INSTIs (n = 62) (see Table 2 and Tables S1-S3, electronic supplementary material, for [M]ANCOVAs and neuropsychiatric scores for the different ARV classes). Limiting our analysis to females (n = 5 for DTG and n = 11 for DTG-naive) did not change these results. In individuals aged ≥ 55 years, BIS-11 scores differed significantly between individuals on DTG (n = 27) and non-DTG regimens (n = 53, F_(3,73)_ = 2.8, *p* = 0.047, η_p_^2^ = 0.10), with lower non-planning impulsivity scores in those on DTG (adjusted mean difference [95%CI] = -− 3.16 (− 5.51 to − 0.8), *p* = 0.0093). After exclusion of individuals on efavirenz (n = 1 for DTG and n = 16 for DTG-naive), we observed a marginal group difference in BIS-11 scores (F_(3,170)_ = 2.7, *p* = 0.045, η_p_^2^ = 0.05), driven by higher motor impulsivity scores in the DTG group (adjusted mean difference [95%CI] = 1.17 [0.13 to 2.22], *p* = 0.027). Within the DTG group, individuals on ABC (n = 59) had lower DASS-42 scores compared to those on alternative backbones (n = 23, F_(3,75)_ = 4.6, *p* = 0.0052, η_p_^2^ = 0.16). Post-hoc analyses showed significantly lower scores for anxiety (adjusted mean difference [95%CI] = -− 3.96 [− 6.16 to − 1.76], *p* = 0.00060), and a trend for lower stress scores (adjusted mean difference [95%CI] = -− .81 [− 6.15 to 0.52], *p* = 0.097) in individuals on DTG-ABC combinations.

**Table 2:** DASS-42, BIS-11 and MATE-Q Results for dolutegravir-treated and dolutegravir-naive individuals

Item		DTG mean (SD)	DTG-naïve mean (SD)	Crude model mean diff (95% CI)	*p*-value	Adjusted model mean diff (95%CI)	*p*-value
DASS-42	Depression	7.2 (6.8)	8.2 (9.3)	− 1.03 (− 3.42 to 1.37)	0.39	− 1.36 (− 3.84 to 1.11)	0.28
	Anxiety	3.9 (4.3)	4.7 (5.4)	− 0.80 (− 2.23 to 0.62)	0.27	− 0.66 (− 2.13 to 0.82)	0.38
	Stress	8.3 (5.9)	9.2 (8.4)	− 0.88 (− 3.02 to 1.26)	0.42	− 1.05 (− 3.27 to 1.17)	0.35
BIS-11	Attentional	16.3 (3.1)	15.8 (3.1)	0.54 (− 0.35 to 1.43)	0.23	0.28 (− 0.62 to 1.19)	0.54
	Motor	21.1 (3.5)	20.1 (3.3)	1.10 (0.13 to 2.07)	0.027	0.95 (− 0.05 to 1.95)	0.062
	Non-planning	24.1 (4.8)	24.5 (5)	− 0.40 (− 1.80 to 1.00)	0.57	− 0.44 (− 1.88 to 1.01)	0.55
Item		n/N (%)	n/N (%)	Crude model OR (95 CI%)	P-value	Adjusted model OR (95% CI)	P-value
Psychiatric scores > cut-off							
DASS-42^*^	Depression	13/82 (15.9)	28/112 (25.0)	0.57 (0.27 to 1.17)	0.13	0.49 (0.22 to 1.06)	0.069
	Anxiety	11/82 (13.4)	18/112 (16.1)	0.81 (0.36 to 1.82)	0.61	0.84 (0.36 to 1.96)	0.69
	Stress	6/82 (7.3)	17/112 (15.2)	0.44 (0.17 to 1.17)	0.10	0.34 (0.12 to 0.96)	0.04
BIS-11^*†*^	Total	14/82 (17.1)	13/112 (11.6)	1.57 (0.69 to 3.54)	0.28	1.43 (0.61 to 3.37)	0.41
MATE-Q substance use							
Active smoking		25/82 (30.5)	33/112 (29.5)	1.05 (0.56 to 1.95)	0.88	0.94 (0.48 to 1.81)	0.85
Heavy alcohol use^*‡*^		10/82 (12.2)	14/112 (12.5)	0.97 (0.41 to 2.31)	0.95	1.05 (0.43 to 2.59)	0.91
Regular substance use^*§*^		27/82 (32.9)	28/112 (25.0)	1.73 (0.93 to 3.22)	0.083	1.47 (0.76 to 2.84)	0.25
	Cannabis	10/82 (12.2)	9/112 (8.0)	1.59 (0.62 to 4.11)	0.34	1.66 (0.6 to 4.56)	0.33
	Opiates^*║*^	1/82 (1.2)	0/112 (0.0)	–	–	–	–
	Cocaine^*║*^	1/82 (1.2)	2/112 (1.8)	–	–	–	–
	Stimulants^*║*^	0/82 (0.0)	1/112 (0.9)	–	–	–	–
	Ecstasy^*║*^	3/82 (3.7)	4/112 (3.6)	–	–	–	–
	Poppers^¶^	12/82 (14.6)	12/112 (10.7)	1.43 (0.61 to 3.36)	0.41	1.23 (0.51 to 2.99)	0.64
	GHB	11/82 (13.4)	11/112 (9.8)	1.42 (0.58 to 3.46)	0.44	1.1 (0.43 to 2.82)	0.84

Mean (SD) DASS-42 and BIS-11 scores, prevalence of scores above the normal cut-off, and prevalence of substance use for DTG-treated and DTG-naive individuals. Data analyzed using univariate analyses of covariance (DASS-42 and BIS-11 scores) or logistic regression models (scores > cut-off and substance use). Age, nadir CD4^+^ cell count and duration of HIV infection were included as covariates in the adjusted models

*Cut-off scores for the DASS-42 are: depression scores > 9, anxiety scores > 7, and stress scores > 14

^†^BIS-11 total scores of ≥ 72 indicate high impulsivity^18^

^‡^Classified according to the CDC definition: for men, ≥ 15 drinks per week and for women, ≥ 8 drinks/week. http://www.cdc.gov/alcohol/faqs.htm#heavyDrinking (page accessed July 1, 2018, Page last reviewed: March 29, 2018, Content source: Division of Population Health, National Center for Chronic Disease Prevention and Health Promotion, Centers for Disease Control and Prevention)

^§^Defined as use of any psychoactive substance (with the exception of alcohol and tobacco) during periods ≥ 1 time per week including ≥ 1 time during the 30 days prior to the study visit

^║^Not statistically analyzed given the low number of events

^¶^Recreational drug class belonging to alkyl nitrites (inhalants)

*BIS-11* Barratt Impulsiveness Scale-1, *DASS-42* Depression Anxiety Stress Scale-42, *DTG* dolutegravir, *GHB* γ-Hydroxybutyric acid, *MATE-Q* Measurements in the Addictions for Triage and Evaluation Questionnaire

In addition to symptoms of mood and impulsivity, we evaluated the frequency of substance use in our study sample. Regular substance use was defined as use of any psychoactive substance (with the exception of alcohol and tobacco) during periods ≥ 1 time per week including ≥ 1 time during the 30 days prior to the study visit. A high proportion of the participants regularly used recreational drugs (58/194 [29.9%]), with cannabis, γ-Hydroxybutyric acid (GHB), and alkyl nitrites (“poppers”) as the most commonly used agents. We observed significant positive associations between regular substance use and depression (*p* = 0.012) and stress scores (*p* = 0.045), and a trend with attentional impulsivity scores (*p* = 0.092) (Table 3). Substance use did not differ between treatment groups (Table 2), nor did it confound our primary analyses (Table S4, Electronic supplementary material for adjusted MANCOVAs with and without substance use). Finally, we found no differences in self-reported headache complaints and concentration disturbances (Table 1).

**Table 3:** Spearman’s correlations between substance use and DASS-42 and BIS-11 scores

Item	Regular substance use		Heavy alcohol drinking		Active smoking	
	ρ	*p*-value	ρ	*p*-value	ρ	*p*-value
DASS-42 total	0.17	0.022	0.030	0.68	0.054	0.45
Depression	0.18	0.012	− 0.0049	0.95	0.093	0.20
Anxiety	0.096	0.18	0.060	0.41	0.099	0.17
Stress	0.14	0.045	0.060	0.41	0.0068	0.93
BIS-11 total	0.099	0.17	− 0.011	0.88	0.18	0.014
Attentional	0.12	0.092	− 0.021	0.77	0.13	0.071
Motor	0.093	0.20	− 0.041	0.57	0.086	0.23
Non-planning	0.056	0.44	0.020	0.79	0.19	0.0073

*BIS-11* Barratt Impulsiveness Scale-1, *DASS-42* Depression Anxiety Stress Scale-42

## Discussion

In the present study, we found a high frequency of depressed and anxious moods and impulsivity in long-term treated PLHIV, which was associated with regular substance use and not with DTG use. Similarly, we observed no differences when comparing DTG with other INSTIs, and INSTIs with non-INSTIs. Our findings are in line with data from clinical trials, which reported relatively few DTG-related neuropsychiatric side effects. Moreover, studies show improvement of neuropsychiatric symptoms after substituting efavirenz for DTG [29–31] and a recent meta-analysis showed no increased risk for suicidality among DTG-treated individuals [32]. However, our findings are in contrast with observational studies, which predominantly focused on individuals who discontinued DTG or other INSTIs. These individuals commonly reported neuropsychiatric symptoms at time of switch [10, 33–35]. In our cohort, a minority of individuals (9% of ever DTG users) had discontinued DTG before, with a very small portion (3% of ever DTG users) potentially related to neuropsychiatric side effects. Our data support that DTG is well tolerated by most individuals. The occurrence of neuropsychiatric side effects in a small subset of DTG-treated individuals, however, cannot be ruled out.

Mechanistically, DTG penetrates the blood brain barrier and may inhibit organic cation transporter 2 (OCT2), which is involved in the transport of serotonin and dopamine [36, 37]. While one study indeed showed that DTG-treated individuals with a genetic variant of the gene encoding for the OCT2 (SLC22A2 C > A) were at higher risk of having abnormal scores of the Symptom Checklist (SCL)-90-R [37], the lack of a control group of DTG-naive PLHIV limits causal inference. Several risk factors have been proposed for DTG-related neuropsychiatric side effects, including older age [11, 34, 38–41], female sex [9, 40, 41], and a backbone containing ABC [9, 33, 41, 42]. In our study, separate analyses in older individuals and females showed similar results, though the latter should be interpreted with caution given the small sample of females. Interestingly, we observed lower rather than higher levels of mood symptoms in individuals on DTG-ABC combinations. This may be caused by channeling due to certain patient characteristics, e.g. by prescribing TDF-containing backbones to PLHIV with cardiovascular disease or hepatitis B, conditions that have also been associated with an increased prevalence of neuropsychiatric symptoms [43, 44]. Still, our observations are in line with several other studies reporting no associations between DTG-ABC use and neuropsychiatric symptoms [11, 40]. In addition, no differences in DTG plasma trough concentrations were found between patients on ABC, rilpivirine, or darunavir [45, 46]. Together, these findings argue against a previously suggested DTG-ABC pharmacokinetic amplification of side effects. Finally, efavirenz co-treatment, which was most prevalent in the DTG-naive group, did not seem to obscure group differences in mood symptoms.

The currently available real life studies on DTG-related neuropsychiatric side effects often relied on spontaneous recordings of psychiatric symptoms in patient files in retrospective designs. The application of standardized validated psychiatric assessment tools is a major strength of the current study, increasing reliability and validity. Using comparable questionnaires, we and others previously observed unexpectedly high levels of neuropsychiatric symptoms among individuals on long-term efavirenz [47–49]. Absence of high symptom levels in individuals on DTG-containing regimens, when applying similar methodology in a sample that was sufficiently powered for detection of small-medium effects, strongly argues against such a phenomenon in DTG.

Another strength of our study is that, by selecting individuals on long-term cART with suppressed viral loads and no acute infections, we reduced the chance of potential HIV-related confounders. Our study has several limitations. First, our study was not primarily designed to assess adverse effects of DTG and we did not examine participants before and after initiation of DTG. Second, we cannot rule out that our study population may have consisted of a selection of people more tolerant to DTG as we excluded individuals who previously discontinued DTG from our analyses. Third, while we used validated questionnaires to assess mood and impulsivity, we did not employ a gold-standard instrument for examining drug-induced neuropsychiatric symptoms in general (including sleep disturbances), like the Scandinavian Society of Psychopharmacology UKU Rating Scale [50]. Fourth, we have limited data on females and non-Caucasian individuals and future research should confirm the tolerability of DTG and generalizability of our findings to these populations.

Despite these drawbacks, we believe that our data, obtained from a sufficiently large patient sample using validated psychiatric tools, supports the safety of dolutegravir in the majority of PLHIV. Still, psychiatric symptoms were common in our population, with scores above the normal cut-off in 26.3% and 13.9% on the DASS-42 and BIS-11 respectively. While these cannot be accounted for by DTG, other factors are likely to contribute. For example, traumatic experiences, stigma, and substance use disorders have been shown to contribute to high levels of mental health problems in PLHIV [51, 52]. Indeed, we found significant associations between regular substance use and depression and stress scores. By working synergistically in promoting behaviors that impede treatment adherence, substance use and depression may also lead to cART discontinuation [53, 54].

Our study highlights the need for proactive screening of mental health problems and substance use in PLHIV, and subsequent referral for adequate psychiatric care if needed. Finally, future studies on neuropsychiatric side effects of ARVs should not only use validated instruments, but also integrate host and lifestyle factors such as substance use.

## Supplementary Information

Below is the link to the electronic supplementary material.

Supplementary file1 (DOCX 107 KB)

## Data Availability

All data were deposited into the publicly accessible DANS EASY archive (URL is being generated and will follow; until then all data are available from the corresponding author upon request).
