# Supplementary material for: Substance use, Unlike Dolutegravir, is Associated with Mood Symptoms in People Living with HIV
Source: AIDS Behav. 2021 Apr 27;25(12):4094–101. doi: 10.1007/s10461-021-03272-2 (PMC8602138; doi:10.1007/s10461-021-03272-2)
Supplement: Supplementary file 1 — Supplementary file1 (DOCX 107 KB) [file 10461_2021_3272_MOESM1_ESM.docx]

**Electronic Supplementary Material**

**Full title:** **Substance use, unlike dolutegravir, is associated with mood symptoms in people living with HIV**

**Table of contents**

Figure S1. Study flowchart 2

Table S1. Mean (SD) DASS-42 and BIS-11 Scores and scores > normal cut-off the Different

ARV Classes 3

Table S2a. Multivariate Analyses of Covariance of DASS-42 4

Table S2b. Multivariate Analyses of Covariance of BIS-11 4

Table S3. DASS-42 and BIS-11 Post-hoc Univariate Analyses of Covariance

Table S4. Adjusted Multivariate Analyses of Covariance of the DASS-42 and BIS-11 with

and without substance use 5

**Figure S1. Study flowchart**

**
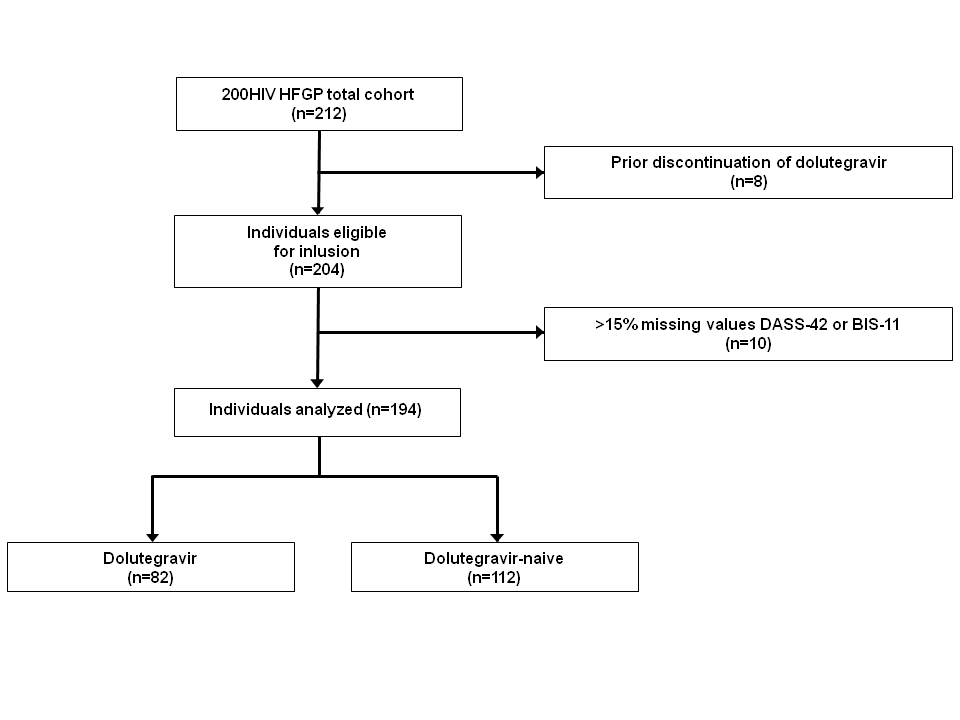
**

*BIS-11, Barratt Impulsiveness Scale-11; DASS-42, Depression Anxiety Stress Scale-42;* *HFGP, Human Functional Genomics Project;*

**Table S1. Mean (SD) DASS-42 and BIS-11 Scores and scores > normal cut-off the Different ARV Classes**

|  |  | **DTG**  **(n=82)** | | **DTG-naive**  **(n=112)** | | **INSTI**  **(n=132)** | | **Non-INSTI**  **(n=62)** | | **RAL**  **(n=35)** | | **EVG**  **(n=14)** | | **DTG-ABC**  **(n=59)** | | **DTG Non-ABC**  **(n=23)** | |
| --- | --- | --- | --- | --- | --- | --- | --- | --- | --- | --- | --- | --- | --- | --- | --- | --- | --- |
| **Item** |  | **Mean (SD)** | **No. (%)** | **Mean (SD)** | **No. (%)** | **Mean (SD)** | **No. (%)** | **Mean (SD)** | **No. (%)** | **Mean (SD)** | **No. (%)** | **Mean (SD)** | **No. (%)** | **Mean (SD)** | **No. (%)** | **Mean (SD)** | **No. (%)** |
| DASS-42 | depression | 7.2 (6.8) | 13 (15.9) | 8.2 (9.3) | 28 (25.0) | 8.0 (8.0) | 29 (22.0) | 7.4 (9.2) | 12 (19.4) | 8.4 (7.8) | 11 (31.4) | 11.8 (13.2) | 5 (35.7) | 6.5 (7.1) | 8 (13.6) | 9 (5.7) | 5 (21.7) |
|  | anxiety | 3.9 (4.3) | 11 (13.4) | 4.7 (5.4) | 18 (16.1) | 4.3 (4.7) | 20 (15.2) | 4.4 (5.5) | 9 (14.5) | 5.1 (5.2) | 6 (17.1) | 5.3 (6.0) | 3 (21.4) | 2.7 (3.3) | 3 (5.1) | 6.8 (5) | 8 (34.8) |
|  | stress | 8.3 (5.9) | 6 (7.3) | 9.2 (8.4) | 17 (15.2) | 8.7 (6.9) | 14 (10.6) | 9.2 (8.5) | 9 (14.5) | 8.5 (6.9) | 4 (11.4) | 11.4 (11.4) | 4 (28.6) | 7.8 (6) | 5 (8.5) | 9.7 (5.7) | 1 (4.3) |
| BIS-11 | attentional | 16.3 (3.1) | - | 15.8 (3.1) |  | 16.3 (3.1) |  | 15.5 (3.2) |  | 15.8 (2.7) |  | 17 (3.6) | - | 16.1 (3.3) | - | 16.9 (2.6) | - |
|  | motor | 21.2 (3.5) | - | 20.1 (3.3) |  | 20.7 (3.5) |  | 20.3 (3.2) |  | 19.6 (3.3) |  | 20 (3.8) | - | 21.5 (3.4) | - | 20.3 (3.8) | - |
|  | non-plan. | 24.1 (4.8) | - | 24.5 (5.0) |  | 24.4 (4.9) |  | 24.2 (4.9) |  | 24.2 (4.9) |  | 25.6 (4.9) | - | 23.9 (4.7) | - | 24.6 (4.9) | - |
|  | total | 61.6 (9.1) | 14 (17.1) | 60.4 (8.6) | 13 (11.6) | 61.3 (8.9) | 20 (15.2) | 60.0 (8.5) | 7 (11.3) | 59.6 (8.0) | 2 (5.7) | 62.6 (9.6) | 3 (21.4) | 61.5 (9.1) | 11 (18.6) | 61.8 (9.3) | 3 (13.0) |

*Mean (SD) scores for the DASS-42 and BIS-11 subscales and number (%) of individuals with scores above the normal cut-off. Cut-off scores for the DASS-42 are: depression scores >9, anxiety scores >7, and stress scores >14.^1^ BIS-11 total scores of ≥72 indicate high impulsivity.^2^*

*ABC; abacavir; BIS-11, Barratt Impulsiveness Scale-1; DASS-42, Depression Anxiety Stress Scale-42; DTG, dolutegravir; EVG; elvitegravir; INSTI; integrase inhibitor; RAL; raltegravir.*

^1^Brown TA, Chorpita BF, Korotitsch W, Barlow DH. Psychometric properties of the Depression Anxiety Stress Scales (DASS) in clinical samples. *Behaviour research and therapy.* 1997;35(1):79-89.

^2^Stanford MS, Mathias CW, Dougherty DM, Lake SL, Anderson NE, Patton JH. Fifty years of the Barratt Impulsiveness Scale: An update and review. *Pers Indiv Differ.* 2009;47(5):385-395.

**Table S2a. Multivariate Analyses of Covariance of DASS-42**

|  |  |  |  |  |  | **Crude model** | | | | **Adjusted model** | | | |
| --- | --- | --- | --- | --- | --- | --- | --- | --- | --- | --- | --- | --- | --- |
| **Selection** | **Group 1** | **N** | **Group 2** | **N** |  | **F** | **df** | **P-value** | **η_p_^2^** | **F** | **df** | **P-value** | **η_p_^2^** |
| **All** | DTG | 82 | DTG-naive | 112 |  | 0.4 | (3,190) | 0.74 | 0.01 | 0.4 | (3,187) | 0.76 | 0.01 |
|  | DTG | 82 | Other INSTI | 49 |  | 1.2 | (3,127) | 0.30 | 0.03 | 0.9 | (3,124) | 0.42 | 0.02 |
|  | INSTI | 132 | Non-INSTI | 62 |  | 1.0 | (3,190) | 0.40 | 0.02 | 0.8 | (3,187) | 0.51 | 0.01 |
|  | ABC | 59 | Non-ABC | 23 |  | 7.1 | (3,78) | 0.00027 | 0.22 | 4.6 | (3,75) | 0.0052 | 0.16 |
| **Age≥55** | DTG | 27 | DTG-naive | 53 |  | 0.3 | (3,76) | 0.86 | 0.01 | 0.2 | (3,73) | 0.87 | 0.01 |
|  | DTG | 27 | Other INSTI | 22 |  | 1.0 | (3,45) | 0.40 | 0.06 | 0.7 | (3,42) | 0.54 | 0.05 |
|  | INSTI | 50 | Non-INSTI | 30 |  | 0.5 | (3,76) | 0.69 | 0.02 | 0.6 | (3,73) | 0.59 | 0.03 |
|  | ABC | 16 | Non-ABC | 11 |  | 3.6 | (3,23) | 0.030 | 0.32 | 2.6 | (3,20) | 0.082 | 0.28 |
| **EFV excl.** | DTG | 81 | DTG-naive | 96 |  | 0.9 | (3,173) | 0.44 | 0.02 | 0.9 | (3,170) | 0.45 | 0.02 |
|  | DTG | 81 | Other INSTI | 49 |  | 1.3 | (3,126) | 0.29 | 0.03 | 1.0 | (3,123) | 0.41 | 0.02 |
|  | INSTI | 130 | Non-INSTI | 46 |  | 1.4 | (3,173) | 0.23 | 0.02 | 1.3 | (3,170) | 0.28 | 0.02 |
|  | ABC | 59 | Non-ABC | 22 |  | 7.0 | (3,77) | 0.00032 | 0.21 | 4.5 | (3,74) | 0.0056 | 0.16 |
| **Females*** | DTG | 5 | DTG-naive | 13 |  | 0.3 | (3,14) | 0.79 | 0.07 | - | - | - | - |
|  | DTG | 5 | Other INSTI | 6 |  | 0.4 | (3,7) | 0.75 | 0.15 | - | - | - | - |
|  | INSTI | 11 | Non-INSTI | 7 |  | 0.4 | (3,14) | 0.73 | 0.09 | - | - | - | - |

*Multivariate analyses of covariance of the DASS-42 subscales: DTG vs. DTG-naive, DTG vs. other INSTI, INSTI vs. non-INSTI, DTG-ABC vs. DTG non-ABC. Subgroup analyses were performed for individuals aged ≥55 years, non-efavirenz containing regimens, and females. Age, nadir CD4^+^ cell count and duration of HIV infection were included as covariates in the adjusted models. Adjusted models with p<0.05 were followed by univariate analyses (Supplemental table 2).*

** Given the small sample size, no adjusted models were applied.*

*ABC, abacavir; DASS-42, Depression Anxiety Stress Scale-42; DTG, dolutegravir; EFV, efavirenz; INSTI, integrase inhibitor; η_p_^2^, partial eta squared, effect size for MANCOVA, which can be interpreted as small effect (0.01–0.05), medium effect (0.06–0.13), and large effect (≥0.14).*

**Table S2b. Multivariate Analyses of Covariance of BIS-11**

|  |  |  |  |  |  | **Crude model** | | | | **Adjusted model** | | | |
| --- | --- | --- | --- | --- | --- | --- | --- | --- | --- | --- | --- | --- | --- |
| **Selection** | **Group 1** | **N** | **Group 2** | **N** |  | **F** | **df** | **P-value** | **η_p_^2^** | **F** | **df** | **P-value** | **η_p_^2^** |
| **All** | DTG | 82 | DTG-naive | 112 |  | 3.1 | (3,190) | 0.029 | 0.05 | 2.1 | (3,187) | 0.11 | 0.03 |
|  | DTG | 82 | Other INSTI | 49 |  | 2.8 | (3,127) | 0.044 | 0.06 | 2.2 | (3,124) | 0.092 | 0.05 |
|  | INSTI | 132 | Non-INSTI | 62 |  | 0.9 | (3,190) | 0.45 | 0.01 | 0.3 | (3,187) | 0.80 | 0.01 |
|  | ABC | 59 | Non-ABC | 23 |  | 1.5 | (3,78) | 0.21 | 0.06 | 0.9 | (3,75) | 0.46 | 0.03 |
| **Age≥55** | DTG | 27 | DTG-naive | 53 |  | 3.3 | (3,76) | 0.025 | 0.11 | 2.8 | (3,73) | 0.047 | 0.10 |
|  | DTG | 27 | Other INSTI | 22 |  | 2.3 | (3,45) | 0.092 | 0.13 | 2.0 | (3,42) | 0.12 | 0.13 |
|  | INSTI | 50 | Non-INSTI | 30 |  | 1.0 | (3,76) | 0.39 | 0.04 | 0.7 | (3,73) | 0.56 | 0.03 |
|  | ABC | 16 | Non-ABC | 11 |  | 0.7 | (3,23) | 0.57 | 0.08 | 0.5 | (3,20) | 0.71 | 0.06 |
| **EFV excl.** | DTG | 81 | DTG-naive | 96 |  | 3.6 | (3,173) | 0.015 | 0.06 | 2.7 | (3,170) | 0.045 | 0.05 |
|  | DTG | 81 | Other INSTI | 49 |  | 2.9 | (3,126) | 0.037 | 0.06 | 2.3 | (3,123) | 0.078 | 0.05 |
|  | INSTI | 130 | Non-INSTI | 46 |  | 0.8 | (3,173) | 0.52 | 0.01 | 0.5 | (3,170) | 0.70 | 0.01 |
|  | ABC | 59 | Non-ABC | 22 |  | 1.2 | (3,77) | 0.32 | 0.04 | 0.8 | (3,74) | 0.51 | 0.03 |
| **Females*** | DTG | 5 | DTG-naive | 13 |  | 0.7 | (3,14) | 0.55 | 0.14 | - | - | - | - |
|  | DTG | 5 | Other INSTI | 6 |  | 0.8 | (3,7) | 0.52 | 0.26 | - | - | - | - |
|  | INSTI | 11 | Non-INSTI | 7 |  | 0.4 | (3,14) | 0.76 | 0.08 | - | - | - | - |

*Multivariate analyses of covariance of the BIS-11 subscales: DTG vs. DTG-naive, DTG vs. other INSTI, INSTI vs. non-INSTI, DTG-ABC vs. DTG non-ABC. Subgroup analyses were performed for individuals aged ≥55 years, non-efavirenz containing regimens, and females. Age, nadir CD4^+^ cell count and duration of HIV infection were included as covariates in the adjusted models. Adjusted models with p<0.05 were followed by univariate analyses (Supplemental table 2).*

** Given the small sample size, no adjusted models were applied.*

*ABC, abacavir; BIS DTG, dolutegravir; EFV, efavirenz; INSTI, integrase inhibitor; η_p_^2^, partial eta squared, effect size for MANCOVA, which can be interpreted as small effect (0.01–0.05), medium effect (0.06–0.13), and large effect (≥0.14).*

**Table S3. DASS-42 and BIS-11 Post-hoc Univariate Analyses of Covariance**

|  |  |  |  | **Crude model** |  | **Adjusted model** |  |
| --- | --- | --- | --- | --- | --- | --- | --- |
| **Selection** | **Item** | **Mean (SD)** | **Mean (SD)** | **Mean difference**  **(95% CI)** | **P-value** | **Mean difference**  **(95% CI)** | **P-value** |
| **All** |  | **DTG-ABC n=59** | **DTG non-ABC n=32** |  |  |  |  |
|  | DASS-42 depression | 6.5 (7.1) | 9 (5.7) | -2.49 (-5.78 to 0.80) | 0.14 | -2.77 (-6.56 to 1.03) | 0.15 |
|  | anxiety | 2.7 (3.3) | 6.8 (5) | -4.05 (-5.94 to -2.15) | <0.0001 | -3.96 (-6.16 to -1.76) | 0.00060 |
|  | stress | 7.8 (6) | 9.7 (5.7) | -1.90 (-4.78 to 0.98) | 0.19 | -2.81 (-6.15 to 0.52) | 0.097 |
| **Age≥55** |  | **DTG-ABC n=16** | **DTG non-ABC n=11** |  |  |  |  |
|  | DASS-42 depression | 5.2 (4.3) | 8.5 (5.8) | -3.35 (-7.36 to 0.66) | 0.098 | -3.03 (-7.24 to 1.19) | 0.15 |
|  | anxiety | 2.6 (2.2) | 7 (4.8) | -4.44 (-7.27 to -1.61) | 0.0035 | -4.66 (-8.06 to -1.27) | 0.0094 |
|  | stress | 6.3 (5.6) | 9.5 (5.4) | -3.14 (-7.6 to 1.31) | 0.16 | -3.25 (-8.11 to 1.62) | 0.18 |
| **Age≥55** |  | **DTG n=27** | **DTG-naive n=53** |  |  |  |  |
|  | BIS-11 attentional | 15.6 (2.7) | 15.4 (3.0) | 0.11 (-1.28 to 1.5) | 0.87 | 0.08 (-1.4 to 1.57) | 0.91 |
|  | motor | 20.2 (3.3) | 20.3 (3.6) | -0.14 (-1.79 to 1.51) | 0.87 | -0.34 (-2.09 to 1.42) | 0.70 |
|  | non-planning | 22.0 (3.2) | 25.2 (4.9) | -3.17 (-5.38 to -0.97) | 0.0053 | -3.16 (-5.51 to -0.8) | 0.0093 |
| **No EFV** |  | **DTG n=81** | **DTG-naive n=96** |  |  |  |  |
|  | BIS-11 attentional | 16.3 (3.2) | 16.0 (3.2) | 0.29 (-0.65 to 1.23) | 0.54 | 0.05 (-1.00 to 0.90) | 0.92 |
|  | motor | 21.2 (3.5) | 19.9 (3.4) | 1.35 (0.32 to 2.37) | 0.010 | 1.17 (0.13 to 2.22) | 0.027 |
|  | non-planning | 24.1 (4.8) | 24.6 (5.2) | -0.55 (-2.04 to 0.94) | 0.47 | -0.61 (-2.14 to 0.92) | 0.43 |
| **No EFV** |  | **DTG-ABC n=59** | **DTG non-ABC n=22** |  |  |  |  |
|  | DASS-42 depression | 6.5 (7.1) | 8.9 (5.8) | -2.36 (-5.72 to 1.00) | 0.17 | -2.68 (-6.5 to 1.14) | 0.17 |
|  | anxiety | 2.7 (3.3) | 6.8 (5.1) | -4.04 (-5.97 to -2.10) | <0.0001 | -3.95 (-6.17 to -1.73) | 0.00069 |
|  | stress | 7.8 (6) | 9.5 (5.7) | -1.70 (-4.64 to 1.24) | 0.25 | -2.69 (-6.03 to 0.65) | 0.11 |

*Post-hoc univariate analyses of covariance following significant multivariate analyses of covariance (Supplemental table 2a and 2b). Age, nadir CD4^+^ cell count and duration of HIV infection were included as covariates in the adjusted models. P-values are Bonferroni-corrected.*

*ABC, abacavir; BIS-11 Barratt Impulsiveness Scale-11; DASS-42, Depression Anxiety Stress Scale-42; DTG, dolutegravir; INSTI, integrase inhibitor*

**Table S4. Adjusted Multivariate Analyses of Covariance of the DASS-42 and BIS-11 with and without substance use**

|  |  |  |  |  |  | **Adjusted model** | | | | **Adjusted model + substance use** | | | |
| --- | --- | --- | --- | --- | --- | --- | --- | --- | --- | --- | --- | --- | --- |
| **Questionnaire** | **Group 1** | **N** | **Group 2** | **N** |  | **F** | **df** | **P-value** | **η_p_^2^** | **F** | **df** | **P-value** | **η_p_^2^** |
| **DASS-42** | DTG | 82 | DTG-naive | 112 |  | 0.4 | (3,187) | 0.76 | 0.01 | 0.5 | (3,186) | 0.67 | 0.01 |
|  | DTG | 82 | Other INSTI | 49 |  | 0.9 | (3,124) | 0.42 | 0.02 | 1.0 | (3,123) | 0.39 | 0.02 |
|  | INSTI | 132 | Non-INSTI | 62 |  | 0.8 | (3,187) | 0.51 | 0.01 | 0.8 | (3,186) | 0.47 | 0.01 |
|  | ABC | 59 | Non-ABC | 23 |  | 4.6 | (3,75) | 0.0052 | 0.16 | 4.6 | (3,74) | 0.0052 | 0.16 |
| **BIS-11** | DTG | 82 | DTG-naive | 112 |  | 2.1 | (3,187) | 0.11 | 0.03 | 2.0 | (3,186) | 0.12 | 0.031 |
|  | DTG | 82 | Other INSTI | 49 |  | 2.2 | (3,124) | 0.092 | 0.05 | 2.2 | (3,123) | 0.092 | 0.051 |
|  | INSTI | 132 | Non-INSTI | 62 |  | 0.3 | (3,187) | 0.80 | 0.01 | 0.3 | (3,186) | 0.85 | <0.01 |
|  | ABC | 59 | Non-ABC | 23 |  | 0.9 | (3,75) | 0.46 | 0.03 | 0.9 | (3,74) | 0.46 | 0.03 |

*Multivariate analyses of covariance of the DASS-42 and BIS-11 subscales: DTG vs. DTG-naive, DTG vs. other INSTI, INSTI vs. non-INSTI, DTG-ABC vs. DTG non-ABC. Age, nadir CD4^+^ cell count and duration of HIV infection were included as covariates in the adjusted models. Substance use had no significantly confounding effect when added to the adjusted model “Adjusted model + substance use.”*

*ABC, abacavir; BIS-11 Barratt Impulsiveness Scale-11; DASS-42, Depression Anxiety Stress Scale-42; DTG, dolutegravir; INSTI, integrase inhibitor; η_p_^2^, partial eta squared, effect size for MANCOVA, which can be interpreted as small effect (0.01–0.05), medium effect (0.06–0.13), and large effect (≥0.14).*
